# Low-energy spontaneous fabrication of quercetin-loaded orange oil nanoemulsion for induction of colorectal cancer cell apoptosis

**DOI:** 10.1186/s12906-026-05424-0

**Published:** 2026-06-17

**Authors:** Ahmed A. Abd-Rabou, Amr E. Edris

**Affiliations:** 1 Hormones Department, Medical Research and Clinical Studies Institute, National Research Center, Cairo, Egypt; 2 Aroma & Flavor Chemistry Department, Food Industries & Nutrition Research Institute, National Research Center, Cairo, Egypt

**Keywords:** Colorectal cancer, Quercetin, Orange oil, Micellar dispersion, nanoemulsion, Cytotoxicity, Apoptosis

## Abstract

**Background:**

Phytochemicals showed potential application as anticancer agents. In the current investigation, quercetin, cold-pressed orange peel oil, and their mixture in their natural state and in micellar dispersion and nanoemulsions were examined for their activity to induce HCT116 and Caco-2 colorectal cancer cells to apoptosis.

**Materials and methods:**

Gas chromatographic analysis was used to characterize orange oil composition. Different solubilization systems were developed in order to incorporate these phytochemicals into a self emulsifyable concentrate that spontaneously forms micellar dispersions and nanoemulsions upon titration into water. Different evaluations were performed to characterize the nanoemulsions. Some biological evaluations including cytotoxicity, apoptosis, protein expression, and reactive oxygen species were also performed.

**Results:**

Gas chromatographic analysis showed that *d*-limonene is the major constituent contained (89%) of orange oil. Systems composed of a single surfactant and a mixture of two surfactants and a cosurfactant were able to incorporate 500 mg quercetin and 5% orange oil in 100 ml nanoemulsions. The particle size of the nanoemulsions ranges between 12 ± 0.09 nm and 27 ± 0.1 nm, depending on the incorporated phytochemical, with z-potential value in the range of -0.9 mV. Encapsulation efficiency for both quercetin and orange oil was > 93% and the release from the formulations exhibited a controlled, time-dependent profile for both quercetin and orange oil. Quercetin and orange oil in their natural state showed cytotoxic and apoptotic activity against the tested colorectal cancer cells, which is enhanced upon combining these phytochemicals. The micellar dispersions and nanoemulsion form of these phytochemicals showed enhanced cytotoxicity, apoptotic activity, and ROS levels compared to their unformulated natural states indicating the powerful effect of nanoparticles. Moreover, nanoemulsion fabricated with a mixture of two surfactants showed the best cytotoxic and late apoptotic activity through downregulation of CDC25A, PI3k, and Bcl-2 expression levels and upregulation of Bax expression compared to nanoemulsion made using a single surfactant.

**Conclusions:**

Combining quercetin and orange oil in a co-fabricated nanoemulsion could be considered a promising formula that could be examined as an adjuvant to the protocol-prescribed drugs to enhance their activity against colorectal cancer cells.

## Introduction

Colorectal cancer represents the 3rd death cause among cancer patients. Some natural polyphenolic phytochemicals like quercetin can arrest the progression of colon cancer cell proliferation [1] and drive the cells to apoptosis [2, 3]. It can also downregulate growth factor signals that regulate endothelial migration and proliferation of colon cancer cells [4].

The main technical challenge in the application of quercetin in the clinical therapy fields is its poor water solubility and low oral bioavailability. For instance, the water solubility of quercetin is estimated as 0.00215 g/L at 25 °C [5]. That indicates a very sparingly water solubility, which consequently hampers the delivery of quercetin via intravenous injection to an aqueous environment like blood. In addition, its solubility in artificial gastric and intestinal juices is 5.5 µg/mL and 28.9 µg/mL, respectively [6]. That may justify its very low oral bioavailability, which also limits its application in clinical therapeutic applications [7]. For instance, it was found that the absorbed quercetin in humans ranged between 3 and 17% after oral administration of 100 mg/kg quercetin [8].

To overcome this hurdle, some water-based delivery systems like nanoemulsions are developed for enhancing quercetin solubility [9] and bioaccessibility [10]. Nanoemulsions also showed inhibitory activity against the proliferation of colorectal cancer cells [11]. In addition, incorporation of quercetin in the nanoemulsion formula showed inhibitory activity against colorectal HT-29 and HCT-116 cancer cell lines [12].

Based on the above mentioned, the authors in the current investigation are incited to conduct a parallel study from a novel perspective on HCT116 and Caco-2 colorectal cancer cells. That is based on combining quercetin along with another natural co-anticancer phytochemical, like orange-peel oil, in the same nanoemulsion. That is due to the promising potentials of orange oil against colorectal cancer cells [13] which are caused by its high content (up to 95%) of a volatile cyclic monoterpene compound known as d-limonene [14].

To formulate this mixed-phytochemical nanoemulsion, the authors in the current study developed a special solubilization system composed of selected surfactant(s) and cosurfactant that are capable of incorporating quercetin and orange oil mixtures via micellar solubilization, into a homogenous emulsifyable concentrate (EC). Upon titration of that EC into water, the nanoemulsion is spontaneously formed in such a low-energy process that does not require high- energy shear equipment. Different biochemical assays relevant to cytotoxicity, apoptosis, protein expression, and others are applied to support the objective of this investigation.

## Materials and methods

### Materials

Cremophor® RH40 (PEG-40 hydrogenated castor oil, 98%), Tween 80 (polyoxyethylene-20- sorbitan monooleate, 98%), propylene glycol (PG, 99.5%) and d-limonene (97%), α-pinene (≥ 97%), β-myrcene (≥ 90%) and linalool (97%) were purchased from Sigma Aldrich (St. Louis Missouri, USA). MTT (3-(4,5-Dimethylthiazol-2-yl)−2,5-Diphenyltetrazolium Bromide, ≥ 98%), Dimethyl sulfoxide (DMSO, ≥ 99.9%) were purchased from Sigma Aldrich (St. Louis Missouri, USA). RPMI 1640 medium, fetal bovine serum, streptomycin and penicillin, annexin V FITC and propidium iodide (PI) were purchased from (ThermoFisher Scientific, USA). Nitric oxide (NO) and Nitric oxide synthase (NOS2) were purchased from (Wuhan Fine Biotech Co., China). PI3k, CDC25A, Bax, and Bcl-2 kits were purchased from (Sunlong Biotech Co., Ltd). Cold- pressed orange (*Citrus sinensis*) peel oil (100% pure) belong to the Valencia type was obtained from El-Marwa food industries, Egypt. Sunflower oil was purchased from the local market in Egypt.

### Characterization of orange peel oil using gas chromatographic analysis

PerkinElmer Autosystem XL Gas Chromatograph (USA) equipped with a flam ionization detector (GC-FID) was used for that purpose. The oven was programmed to initial and final temperatures of 50 ℃, 220 ℃ respectively, at a rate of 3 ℃/minute, without heating ramps. The injector and FID detector temperatures were 220 °C and 230 °C respectively. Helium was used as carrier gas at a flow rate 1 ml/minute. Separation of volatile components took place using a Carbowax capillary column (60 m × 0.32 mm internal diameter) with a film thickness of 0.25 µm. Identification of d-limonene and other minor terpenes in orange oil took place by comparing their retention time with authentic samples.

### Fabrication of micellar dispersions and nanoemulsions

Three main groups of water-based micellar dispersion and nanoemulsions of quercetin, orange peel oil and their mixture were fabricated (Table 1), using the low-energy spontaneous emulsification technique [15]. The necessary modifications were made to the technique to be able to incorporate a mixture of quercetin powder and orange oil into a stable and homogenous nanoemulsion. Each of the three groups contained two (or more) sub-groups that differed from one another in the composition of surfactant(s). The composition and concentrations of the ingredients used for fabrication of all subgroups were illustrated in Table 1. The details of fabrication of the micellar dispersions and nanoemulsion took place as follows:

**Table 1:** Composition of the different groups and subgroups of quercetin micellar dispersion and orange oil nanoemulsions

Groups	Subgroups	Surfactant	Cosurfactant (PG, %)	Quercetin	Orange oil (%)	Sunflower oil (%)*	DMSO	Water
Group1 Quercetin micellar dispersion	F1 (test)	Cr (5%)	1	500 mg	–	–	–	to 100 ml
	F2 (test)	Cr:T80 (2:1, 5%)	1	500 mg	–	–	–	to 100 ml
	F1	Cr (10%)	2	500 mg	–	–	–	to 100 ml
	F2	Cr:T80 (2:1, 10%)	2	500 mg	–	–	–	to 100 ml
	C1 (control)	–	–	500 mg	–	–	100 ml	–
Group2 Orange oil nanoemulsion	F3	Cr (10%)	2	–	5	1	–	to 100 ml
	F4	Cr:T80 (2:1, 10%)	2	–	5	1	–	to 100 ml
	C2 (control)	–	–	–	5	1	100 ml	–
Group3 Orange oil-quercetin nanoemulsions	F5	Cr (10%)	2	500 mg	5	1	–	to 100 ml
	F6	Cr:T80 (2:1, 10%)	2	500 mg	5	1	–	to 100 ml
	C3 (control)	–	–	500 mg	5	1	100 ml	–

All percentages are weight %

Cr: Cremophor® RH40; T80: Tween80; PG: Propylene glycol

F1 (test), F2 (test): Micellar solutions of quercetin formulated using single surfactant, and mixed surfactants, respectively, at 5%

F1, F2: Micellar solutions of quercetin formulated using single surfactant, and mixed surfactants, respectively at 10%

F3, F4: Orange oil nanoemulsions formulated using single surfactant, and 2 mixed surfactants, respectively at 10%

F5, F6: Orange oil-quercetin mixed nanoemulsion formulated using single surfactant, and 2 mixed surfactants, respectively at 10%

^*^Ostwald ripening inhibitor

#### Fabrication of group (1): quercetin micellar dispersions (F1, F2)

**Fig. 1:**
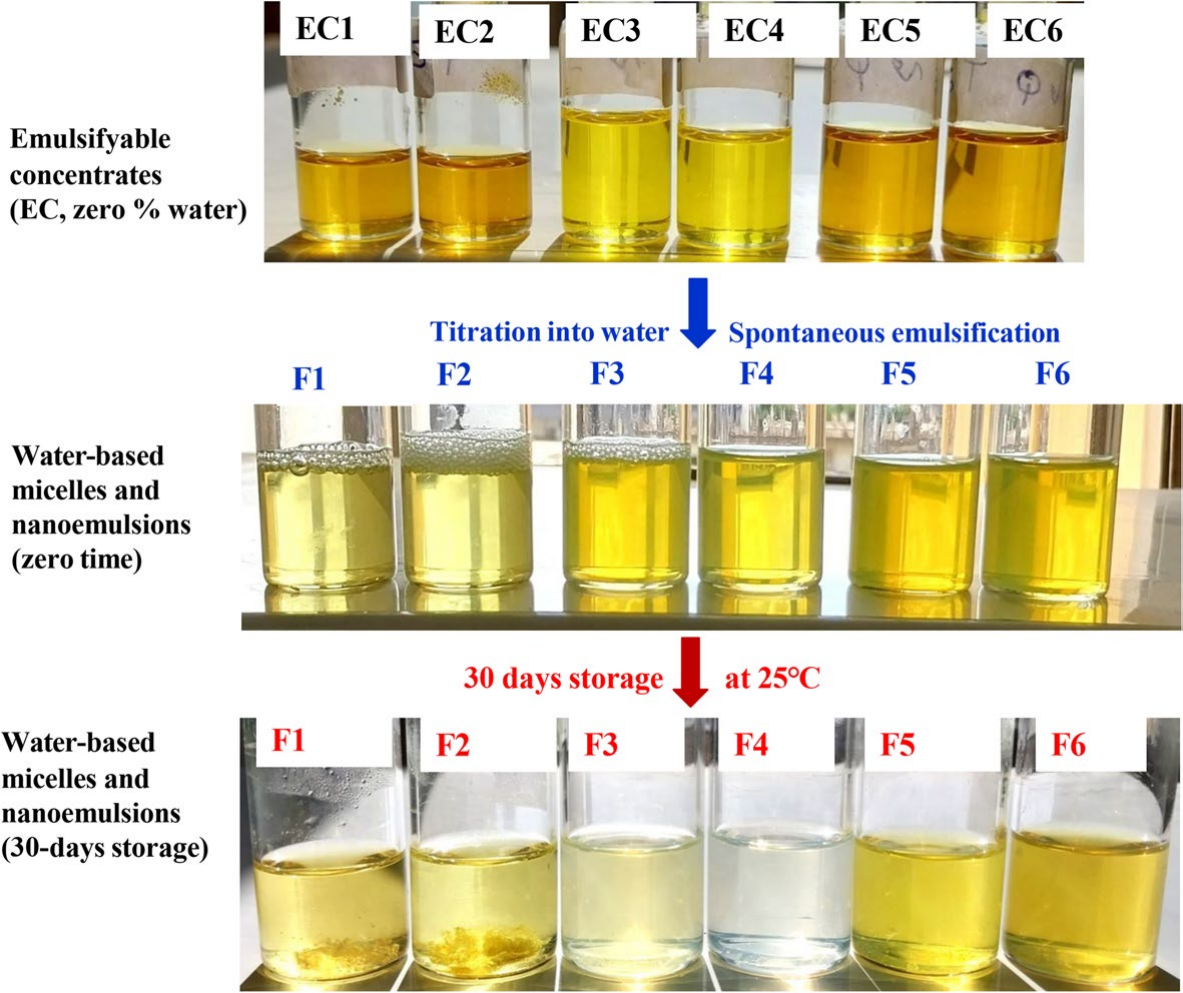
Fabrication and stability of the different quercetin micellar dispersion and orange oil nanoemulsions at 25℃

Four solubilization systems, namely, F1 (test), F2 (test), F1, and F2, were prepared by mixing a single surfactant (Cremophor) or a mixture of surfactants (Cremophor and Tween 80 at a 2:1 weight ratio) with the cosurfactant (propylene glycol, PG) at different concentrations as indicated in Table 1. The ingredients of the solubilization systems were mixed for 2 min at room temperature using a vortex (or mechanical stirrer) until they became homogenous. Then, quercetin powder was added to each solubilization system with warming at 45 ℃ for 1–2 min, followed by mixing till the whole amount of quercetin powder dissolve completely in each solubilization system to give a clear orange-brown colored solution of quercetin emulsifyable concentrates (EC1 and EC2, Fig. 1, Top photos). To initiate the formation of quercetin micellar dispersions, each (EC) was titrated dropwise into water with continuous stirring using a magnetic bar to get spontaneously a transparent water-based quercetin micellar dispersion (F1 and F2, Fig. 1, Middle photos).

A control sample denoted as (C1) was prepared for biological evaluation by dissolving the same quantity of quercetin (500 mg) in 100 ml of an organic solvent like dimethyl sulfoxide (DMSO, Table 1).

#### Fabrication of group (2): orange oil nanoemulsions (F3, F4)

Two solubilization systems were prepared by the same procedure applied above with the replacement of quercetin with 5% cold-pressed orange oil and 1% sunflower oil as Ostwald ripening inhibitor [15] (Table 1). The obtained orange oil emulsifyable concentrates (EC3 and EC4, Fig. 1) were titrated into water under the same conditions as above to get water-based orange oil nanoemulsions (F3 and F4, Fig. 1). These formulations are considered unloaded nanoemulsions (i.e., without quercetin). A control sample denoted as (C2) was also prepared for biological evaluation by adding 5 g of orange oil and 1 g of vegetable oil to 100 ml DMSO, (Table 1).

#### Fabrication of group (3): quercetin-orange oil mixed nanoemulsions (F5, F6)

Two more solubilization systems were also prepared by the same procedure applied above, where quercetin was first dissolved completely in the surfactant(s)-PG solubilization systems, then 5% orange oil and 1% sunflower oil was added to that system to give quercetin-orange oil-mixed emulsifyable concentrates (EC5 and EC6, Fig. 1). Each (EC) was then titrated into water to get quercetin-orange oil mixed nanoemulsions (F5 and F6, Fig. 1). A control sample denoted as (C3) was prepared for biological evaluation by adding 500 mg of quercetin, 5 g of orange oil, and 1 g of vegetable oil to 100 ml DMSO (Table 1).

### Characterization of nanoemulsions

#### Visual inspection and physical stability

The general appearance of nanoemulsion samples was visually monitored during a storage period of 30 days at room temperature (25℃ ± 2). The samples were placed in transparent screw-caped glass vials and stored upright undisturbed in a closed cupboard resting on the bench, in order to prevent photooxidation, at room temperature. All formulations were inspected visually on a daily basis, for detection of possible quercetin precipitation or orange oil separation. In addition, the physical stability at 37 ℃ for 24 h was also observed by the same way.

#### Particle size analysis

The particle size of the different formulas was measured after an equilibration period of 24 h after formulation. The dynamic light scattering instrument Zetasizer (Nano-ZS model ZEN3600, Nanoseries, Malvern Instruments, UK) was used for particle sizing, which was done at room temperature (25℃ ± 2), at a fixed angle of 173°. Before measurement, all formulas were filtered through a 0.20 µm single-use syringe filter unit (Minisart®, Sartoius Stedium Biotech GmbH Germany) to remove impurities. Each sample was diluted before measurement with distilled water to only 0.05% to prevent multiple scattering. Sizes quoted are the *z*-average mean of the droplet’s hydrodynamic diameter (nm) obtained from 6 measurements for each sample (2 duplicate × 3 measurements each). The particle size distribution curves are plotted from dynamic light scattering data as the average of duplicate samples.

#### Zeta potential assessment

The surface charge (zeta potential, mV) of all formulations was measured using the electrophoresis and dynamic light scattering instrument Zetasizer (Nano-ZS). Zeta potential was calculated from the measurement of the electrophoretic mobility of particles in an applied oscillating electric field using laser Doppler velocimetry. All measurements were done in triplicate at 25 ℃ and the values reported were mean ± S.D.

#### Transmission Electron Microscopy (TEM)

Particle morphology of the formulated nanoemulsions was examined using TEM (Philips CM-10 FEI, In., Hillsboro, OR, USA). One drop of the nanoemulsion was loaded on a Formvar-coated copper grids and left to dry. Then the samples were stained using 2% w/v uranyl acetate as a negative staining agent. Image capture and analysis was done using Digital Micrograph and Soft Imaging Viewer software (Electron Microscope Unite services), NRC, Egypt.

#### Encapsulation Efficiency (EE%) measurement

The encapsulation efficiency (EE%) of formulations F1-F6 was determined through a dialysis method using a membrane with a molecular weight cut-off of 20 kDa (Spectrum Laboratories, USA). This method was employed to eliminate any free quercetin and orange oil that were not encapsulated. The quantification of both free and encapsulated forms of quercetin and orange oil was carried out using UV-based spectroscopy with a variable wavelength detector (BMG Labtech, Germany). Quercetin showed characteristic UV absorption peak at 370 nm, while cold-pressed orange oil exhibited absorption at 330 nm. The dialysis system was kept at a constant temperature of 25 °C using a thermostat. The EE% was calculated using the formula:

$$\mathrm{R}\mathrm{e}\mathrm{l}\mathrm{e}\mathrm{a}\mathrm{s}\mathrm{e} (\%) = [\mathrm{R}\mathrm{e}\mathrm{l}\mathrm{e}\mathrm{a}\mathrm{s}\mathrm{e}\mathrm{d} \mathrm{o}\mathrm{i}\mathrm{l} (\mathrm{o}\mathrm{r} \mathrm{q}\mathrm{u}\mathrm{e}\mathrm{r}\mathrm{c}\mathrm{e}\mathrm{t}\mathrm{i}\mathrm{n})/\mathrm{T}\mathrm{o}\mathrm{t}\mathrm{a}\mathrm{l} \mathrm{o}\mathrm{i}\mathrm{l} (\mathrm{o}\mathrm{r} \mathrm{q}\mathrm{u}\mathrm{e}\mathrm{r}\mathrm{c}\mathrm{e}\mathrm{t}\mathrm{i}\mathrm{n})] \times 100$$

### In vitro release studies

The in vitro release of quercetin and orange oil was studied using the dialysis technique. The dialysis bag was first rinsed with phosphate-buffered saline (PBS) to remove any preservatives, and then filled with 3 mL of quercetin or orange oil-loaded formulations (F1-F6) suspended in PBS containing 20% ethanol at pH 7.4. The addition of ethanol prevents clumping and ensure a more consistent release. The release study was conducted over a period of 24 h, with 3 mL aliquots collected at specific intervals, replaced with fresh medium, and analyzed using UV-based spectroscopy (BMG Labtech, Germany). Quercetin showed characteristic UV absorption peak at 370 nm, while orange oil exhibited absorption at 330 nm.

### Cancer cell cultivation

The colorectal cancerous HCT116 and Caco-2 cell lines were purchased from American Type Culture Collection (ATCC) supplied from VCSERA and cultivated in RPMI 1640 medium (ThermoFisher, USA) supplemented with 10% heat-inactivated fetal bovine serum (Gibco), 100 U/mL streptomycin, and 100 U/mL penicillin (ThermoFisher, USA) at 37 °C in a humidified 5% CO_2_ atmosphere.

### Cell proliferation assay

Colorectal cancer HCT116 and Caco-2 cells were seeded in 96-well plates at a density of 1 × 10^4^ cells/well and incubated for 24 h. Then, they subsequently treated with 0, 20, 40, 60, 80, and 100 µg/ml of all formulations (F1-F6) and control samples (C1-C3). We used DMSO only in C1-C3 to solubilize quercetin and orange oil. The final concentrations of DMSO used in the cytotoxicity were 0.1% to 0.5% for the different dosages. The 0.0 µg/ml represents the untreated cells, which received only PBS over the medium. After that, the cells were incubated with 1 mg/ml of MTT reagent at 37 ℃ for 4 h and then it was discarded. The formed formazan crystals were dissolved using 100 ml of DMSO, followed by incubation and shaking. Finally, colorimetric analysis using a multiplate reader was measured at 540 nm. The cell proliferation (%) was calculated and compared with control according to the previously reported protocol [16].

### Measurement of the half inhibitory concentration and fold change

The half maximal inhibitory concentrations (IC_50_) values of the formulations, which are the concentrations that inhibit 50% of cell viabilities, were obtained by plotting the percentages of cell viabilities versus the concentrations of the sample using polynomial concentration–response curve fitting models (OriginPro 8 software).

Finally, the fold changes of IC_50_ values of the unformulated controls (C1-C3) dissolved in DMSO relative to each nanoemulsions (F1-F6) were calculated for HCT116 and Caco-2 cells using the equation.

$$\mathrm{F}\mathrm{o}\mathrm{l}\mathrm{d} \mathrm{c}\mathrm{h}\mathrm{a}\mathrm{n}\mathrm{g}\mathrm{e} = {\mathrm{I}\mathrm{C}}_{50 \mathrm{c}\mathrm{o}\mathrm{n}\mathrm{t}\mathrm{r}\mathrm{o}\mathrm{l}} / {\mathrm{I}\mathrm{C}}_{50 \mathrm{n}\mathrm{a}\mathrm{n}\mathrm{o}\mathrm{e}\mathrm{m}\mathrm{u}\mathrm{l}\mathrm{s}\mathrm{i}\mathrm{o}\mathrm{n}}$$

### Apoptosis and necrosis using flow cytometric analysis

Flow cytometry was used to detect the early and late apoptotic cell distributions and healthy populations in addition to necrotic cells. Colorectal cancer HCT116 and Caco-2 cells were seeded at a density of 1 × 10^6^ cells and incubated for 24 h. Cells were treated with the IC_50_ of the chosen subgroups (F2, F4, and F6) only, beside the control (C1, C2 and C3) as shown in Table 2 and cultivated for 24 h incubation. The choice of these three subgroups for the apoptosis and necrosis study will be justified in details in the results and discussion.

**Table 2:** Particle size, polydispersity index, zeta potential, and encapsulation efficiency of micellar dispersions and nanoemulsions

Formulation	Size (nm)	PDI	Zeta potential (mV)	Encapsulation efficiency	
				Quercetin	Orange oil
F1	14.0 ± 0.02	0.08 ± 0.00	−0.84 ± 0.00	93.84 ± 3.5	-
F2	12.3 ± 0.09^a^	0.08 ± 0.00	−0.92 ± 0.00	97.5 ± 2.4^a^	-
F3	25.9 ± 0.09^a^	0.10 ± 0.00	−0.90 ± 0.001	-	94.98 ± 3.1
F4	26.8 ± 0.2^a^	0.08 ± 0.00	−0.91 ± 0.001	-	95.64 ± 2.8
F5	27.1 ± 0.1^a^	0.09 ± 0.00	−0.92 ± 0.00	96.9 ± 4.5^a^	97.38 ± 3.1^a^
F6	24.5 ± 0.1^a^	0.09 ± 0.00	−0.95 ± 0.00	93.27 ± 2.6	98.41 ± 4.9^a^

F1-F6: refer to footnote of Table 1

PDI: polydispersity index

^a^represents significant difference when comparing each formulation with F1 (*P <* 0.05)

After incubation, all cells were stained with Annexin V FITC and propidium iodide (PI) (Thermo Scientific, USA). Early and late apoptosis as well as necrosis of the treated cells versus untreated cells were analyzed by flow cytometer (Beckman Coulter Instrument, USA). 25,000 events were recorded per sample. Cell distributions were measured using Flow Cytometry and analyzed using its software.

### Measurements of ROS markers

#### Nitric oxide (NO)

Both colorectal HCT116 and Caco-2 cancerous cells were cultured in 96-well plates at a density of 1 × 10^4^ cells/well. On the second day, the IC_50_ dosages of the proposed treatments were added to the media. Nitrate reductase was first used to convert nitrate to nitrite. Then, Griess reagent was used to convert nitrite to a deep purple azo compound. The amount of the azo chromophore accurately reflected the NO amount in the samples. Finally, optical density was measured at OD 540 nm using the microplate reader (BMG Labtech, Germany).

#### Nitric oxide synthase (NOS2)

NOS2 enzyme activity of HCT116 and Caco-2 cells up on treatments with C1-C3 and F2, F4, and F6 was measured using an ELISA kit purchased from (Wuhan Fine Biotech Co., China). This kit was based on the Competitive-ELISA detection procedure. The provided microtiter plate has been pre-coated with target. During the reaction, the target in the sample or standard competes with a fixed amount of target on the solid phase supporter for sites on the Biotinylated Detection Antibody specific to the target. Excess conjugate and unbound sample or standard were washed from the plate, and HRP-Streptavidin (SABC) was added to each microplate well and incubated. Then TMB substrate solution is added to each well. The enzyme–substrate reaction is terminated and the color change is measured spectrophotometrically at a wavelength of 450 nm. The concentration of target in the samples is then determined by comparing the OD of the samples to the standard curve.

### Measurements of apoptotic expression levels

The protein expression levels of the PI3k, CDC25A, Bax, and Bcl-2 were assessed after treating colorectal cancer HCT116 and Caco-2 cell lines with the IC_50_ dosage of the subgroups (F2, F4, F6) and controls (C1, C2, C3) using ELIZA kits purchased from (Sunlong Biotech Co., Ltd). The protocols of the enzyme–substrate reaction were followed according to the manufacturer's manual. The color change after the reaction was measured spectrophotometrically at a wavelength of 450 nm. The concentration of target in the samples is then determined by comparing the absorbance of the samples to the standard curve.

### Statistical analysis

A one-way ANOVA was used for comparison between groups. Significant difference (*P <* 0.05) and high significant difference (*P <* 0.01) compared to control. Results were expressed as mean ± standard error (SE).

## Results

Fabrication and characterization of the micellar dispersion, nanoemulsions and pure orange oil.

**Fig. 2:**
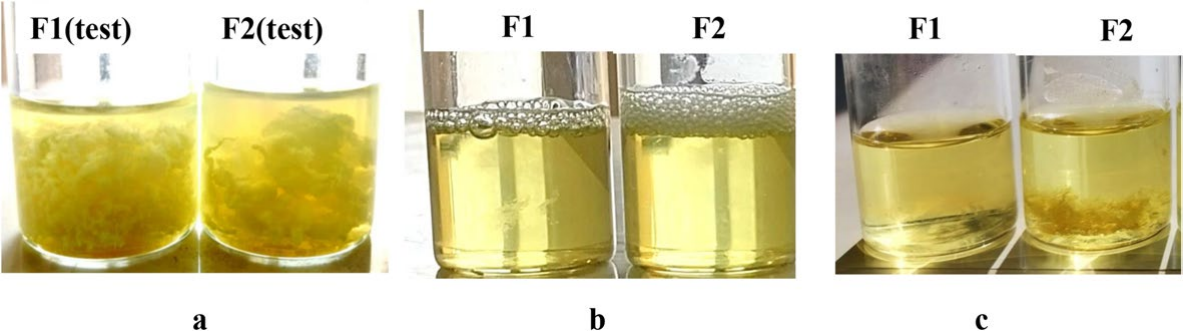
Effect of surfactant(s) and cosurfactant (PG) concentrations on the fabrication and stability of quercetin micellar dispersion. **a** 5% surfactant(s) and 1% PG. **b** 10% surfactant(s) and 2% PG, zero time. **c** 10% surfactant(s) and 2% PG, after 1 week storage period at room temperature

Quercetin powder (500 mg) was first fabricated in two test micellar dispersions, namely, subgroups F1(test) and F2(test). They are composed of 5% surfactant(s) and 1% cosurfactant (PG) and water (Table 1). Results of the fabrication of the test solutions F1(test) and F2(test) showed that quercetin powder was precipitated from both micellar dispersions immediately after titrating their corresponding emulsifyable concentrates (EC) into water (Fig. 2a). Therefore, quercetin formulations were re-fabricated (sub-groups F1 and F2) after gradual increase of the amount of surfactant(s) and PG until reaching an optimum concentration of 10% and 2%, respectively (Table 1). That led to a better formation of transparent water-based quercetin micellar dispersions, as shown in Fig. 2b. Based on this result, all the rest of the other nanoemulsions in group 2 (F3, F4) and group 3 (F5, F6) were fabricated using this optimized surfactant (10%) and PG (2%) concentrations, as shown in Table 1.

**Fig. 3:**
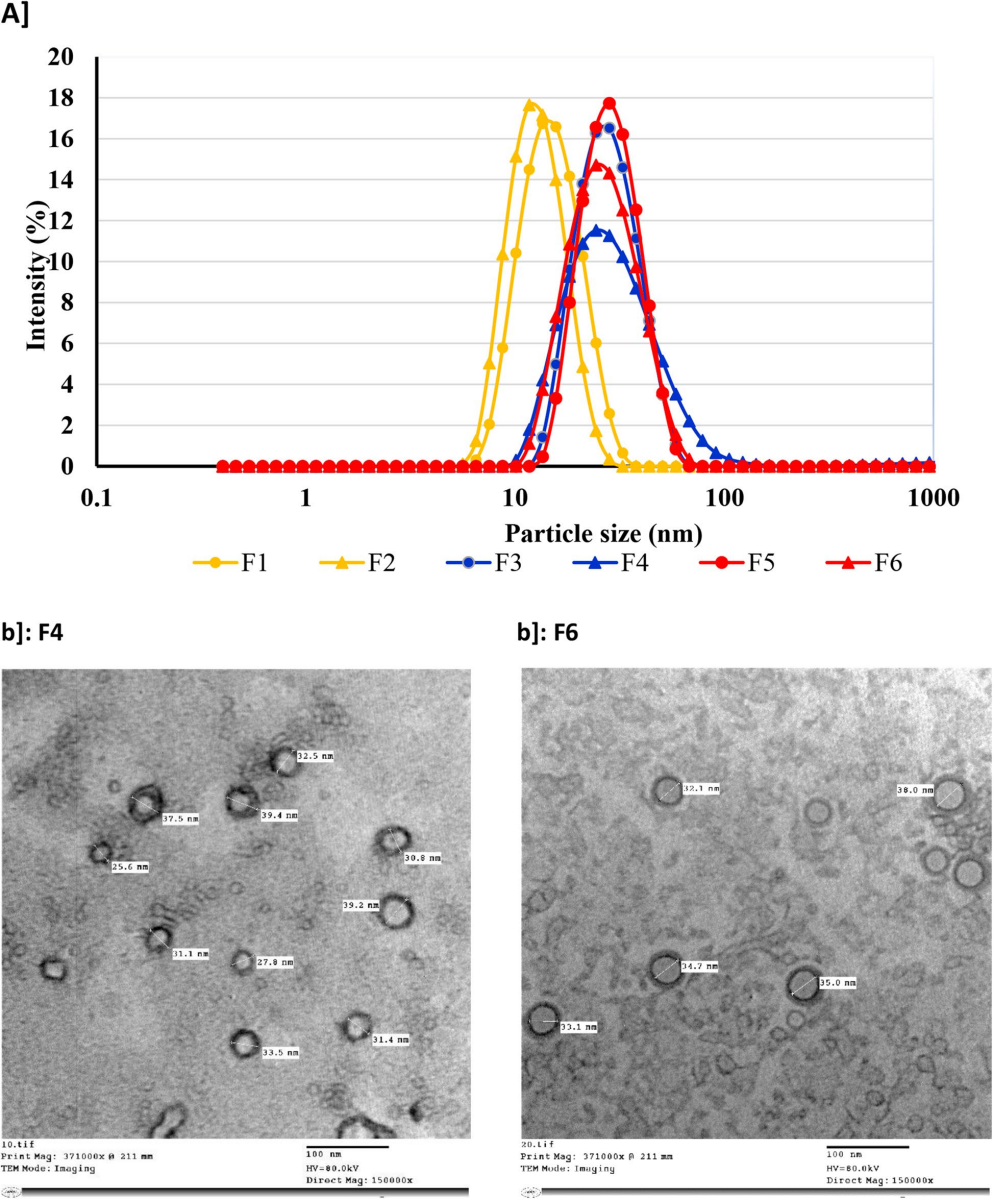
Particle size analysis (**a**) and TEM images (**b**) of the fabricated nanoemulsions

The particle size analysis of quercetin in the micellar solution (F1 and F2) showed that the z-average of quercetin nanoparticles ranged between 14 ± 0.02 nm and 12 ± 0.09 nm, respectively, with monomodal distribution and polydispersibility index, (PDI) 0.08 (Fig. 3a). The Zeta-potential of F1 and F2 were −0.84 ± 0.001 mV and −0.92 ± 0.001 mV (Table 2).

The physical stability of the two aqueous quercetin micellar dispersions was examined for 30 days; however, after only 1 week, formula (F2) was found to be unstable, where some quercetin precipitations were detected (Fig. 2c). On the other hand, formula (F1) was stable during the week, indicating that only a single surfactant like Cremophor at 10% has the necessary hydrophilic-lipophilic balance (HLB) and affinity to quercetin to keep it totally solubilized in the nanoemulsion. However, at the end of the 30-day storage period, fewer quercetin precipitations were detected in F1, as seen in Fig. 1, (bottom).

**Fig. 4:**
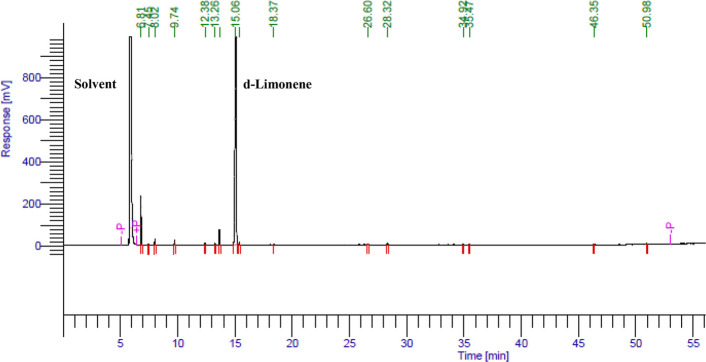
Gas Chromatogram of cold-pressed orange peel oil

Before the fabrication of orange peel oil nanoemulsions (Group 2, Table 1), the pure oil was first subjected to characterization using gas chromatographic analysis (Fig. 4) in order to determine mainly the percentage of d-limonene. Results indicated that this compound comprises 89% of the oil composition. In addition, some other minor components were identified as α-pinene (5.7%), β-myrcene (2.2%), and linalool (0.3%).

After characterization, orange oil is fabricated in two nanoemulsions (Group 2) denoted as (F3 and F4, Table 1) which differ from one another in the composition of surfactant(s). These formulations are considered unloaded nanoemulsions (i.e., without quercetin). Results showed that both nanoemulsions were yellow transparent dispersions (Fig. 1, middle photo) with z-average particle size ranged between 25.9 ± 0.09 nm to 26.8 ± 0.2 nm and monomodal size distribution with PDI ~ 0.1 (Fig. 3a). Zeta-potential of F3 and F4 were −0.90 ± 0.001 mV and −0.91 ± 0.001 mV, (Table 2). The physical stability study at 25℃ for a 30-day storage period indicated that orange oil nanoemulsions (F3 and F4) were stable without detection of any orange oil separation (Fig. 1, bottom). The same physical stability was also maintained after storage at 37℃ for 24h.

Regarding group (3), quercetin was incorporated along with cold-pressed orange oil in the same nanoemulsion formula (F5 and F6, Table 1). Figure 1, middle photo, showed that both nanoemulsions were yellow and transparent. The average particle size ranged between 27.1 ± 0.1 nm and 24.5 ± 0.1 nm, respectively, with monomodal distribution and PDI 0.09 (Fig. 3a). Zeta-potential of F5 and F6 were −0.92 ± 0.001 mV and −0.95 ± 0.001 mV, (Table 2).

After a 30 day-storage period at room temperature, nanoemulsions F5 and F6 were found to be physically stable with no quercetin precipitations or orange oil separation (Fig. 1, bottom). Unlike orange oil nanoemulsions (F3, F4) which turned colorless after storage, the yellow color of F5 and F6 was retained. The same physical stability was also maintained after storage at 37℃ for 24h.

The morphology of quercetin-free orange oil nanoemulsion (F4) and its corresponding quercetin-loaded orange oil nanoemulsion (F6) was studies using TEM. Results indicated that the particles are spherical and homogenous (Fig. 3b), with a diameter correlate well with the results obtained from the Zetasizer measurements (Fig. 3a), with few nanometers difference. The choice of F4 and F6 among other formulations for TEM evaluation is revealed in the discussion section.

### Encapsulation Efficiency (EE%)

All formulations, right after formulation (zero time), exhibited high encapsulation efficiency (> 93%) for both quercetin and orange oil (Table 2). Quercetin-loaded formulations (F1, F2, F5, F6) showed EE% values ranging from 93.27% to 97.5%, with F2 demonstrating the highest encapsulation (97.5 ± 2.4%), followed by F5 (96.9 ± 4.5%), F1 (93.84 ± 3.5%), and F6 (93.27 ± 2.6%), as shown in (Table 2). For orange oil-loaded formulations (F3, F4, F5, F6), the EE% reached its highest value (98.41%) for F6 and the lowest (94.98%) was for F3 (Table 2).

### In vitro release study

**Fig. 5:**
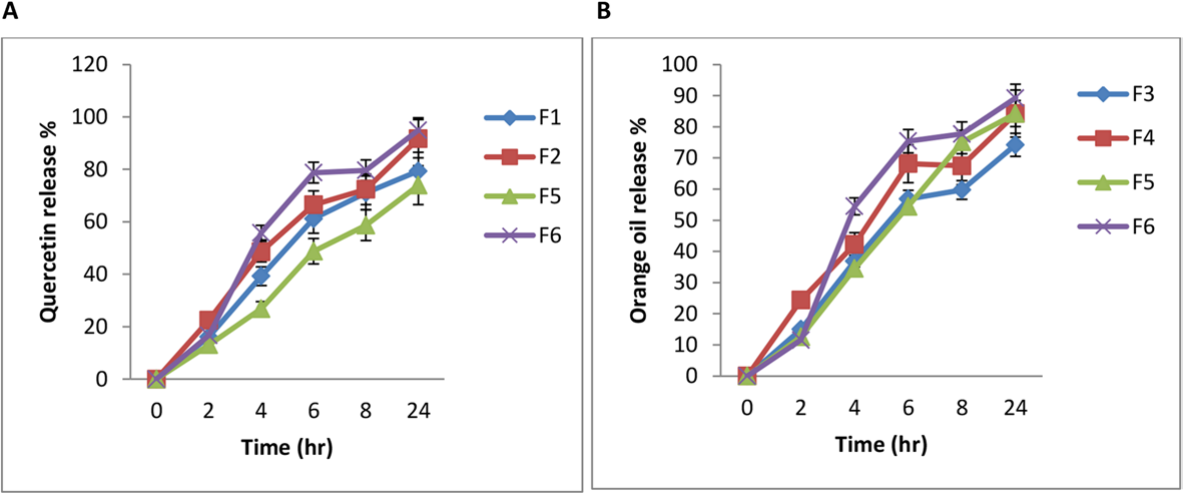
In vitro release profiles of quercetin (**a**) and orange oil (**b**) from the fabricated micellar solution and nanoemulsions

In vitro release study showed that quercetin release ranged from 73.96 ± 3.70% (F5) to 94.93 ± 4.75% (F6), (Fig. 5a). On the other hand, orange oil release ranged from 74.37 ± 3.72% (F3) to 89.25 ± 4.46% (F6) at 24 h (Fig. 5b). Among all formulations, F6 consistently demonstrated the highest release for both encapsulated compounds, indicating superior release performance, as illustrated in (Fig. 5a, b).

### Biological evaluations of micellar dispersions and nanoemulsions against colorectal cancer cells

All the formulated sub-groups (F1-F6) and control samples (C1-C3) are subjected to different evaluations to test their inhibitory activity against HCT116 and Caco-2 colorectal cancer cell lines. These evaluations include cytotoxicity, apoptosis, reactive oxygen species, and protein expression.

#### Cytotoxicity evaluation of the unformulated quercetin and orange oil

**Fig. 6:**
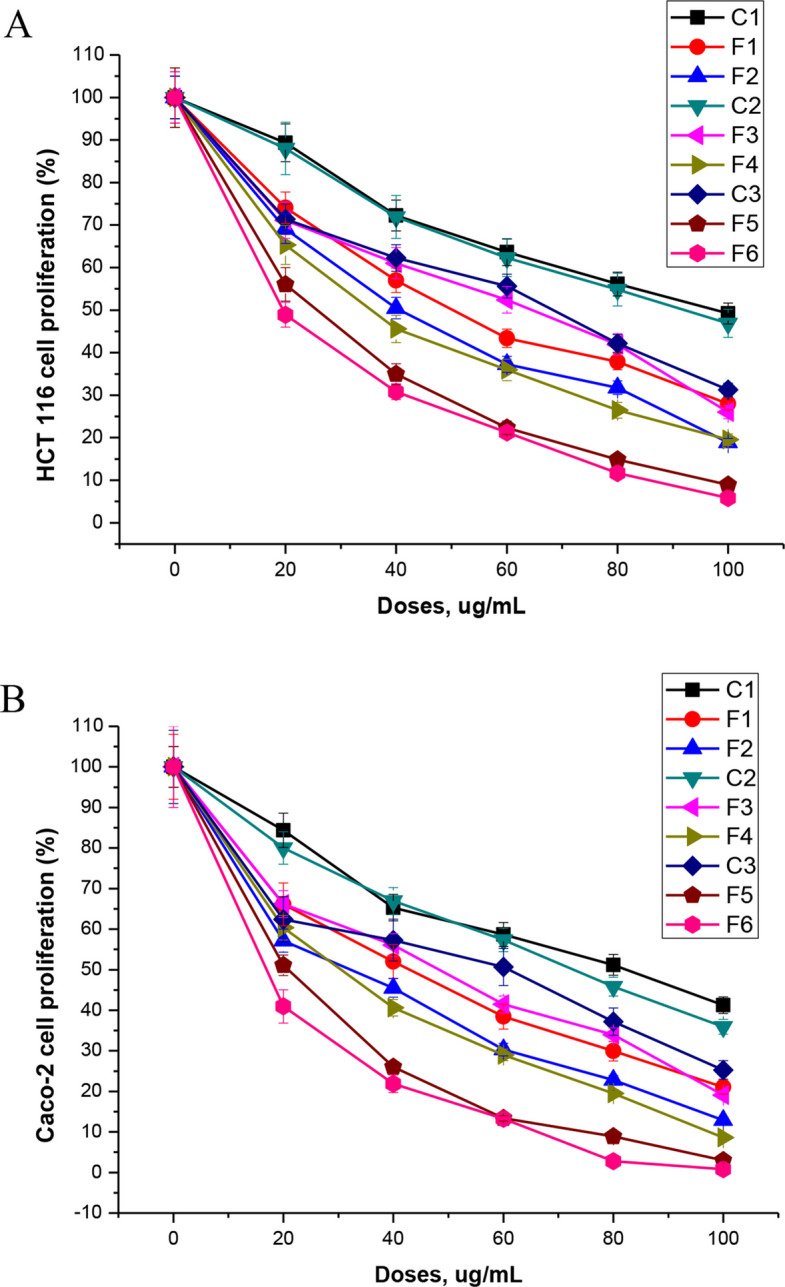
Anti-proliferative activity of different groups of nanoemulsions against (**a**) human colorectal HCT116 and (**b**) Caco-2 cancerous cell lines after 24 h incubation period at 37 °C (*n =* 3)

Control samples (C1-C3) represent the natural unformulated quercetin, orange oil, and their mixture, respectively, dissolved in DMSO (Table 1). It is important to note that the maximum concentration of DMSO used for cytotoxicity evaluation of free quercetin and orange oil was 0.5% which is a safe dose for cell culture. These pure compounds showed dose-dependent cytotoxic activity against both colorectal cancer cell lines (Fig. 6a, b). At the highest tested dose (100 µg/ml), quercetin (C1), orange oil (C2) and quercetin-orange oil mixture (C3) inhibited HCT116 cell proliferation to 49.23%, 46.89% and 31.25%, respectively (Fig. 6a). This result demonstrates the anti-colorectal cancer activity of quercetin and orange oil, especially when they are combined together.

Beside quercetin, natural unformulated orange peel oil in DMSO (C2, Table 1) also demonstrates antiproliferative activity against HCT116 cells with an inhibition value of 46.89% compared to 49.23% for quercetin (Fig. 6a).

Combining quercetin with orange oil in DMSO (control C3, Table 1) led to an enhanced inhibitory activity against the proliferation of HCT116 colorectal cancer cells to reach only 31.25% (Fig. 6a). That clearly demonstrates the synergistic inhibitory effect of a mixture of quercetin and orange oil on the proliferation of cancer cells.

Regarding Caco-2 colorectal cell line (Fig. 6b), the cytotoxicity of unformulated quercetin, orange oil and their mixture (C1-C3) against these cells was illustrated in (Fig. 6b). The results showed the same trend that we described before for HCT116 cells, where the highest inhibition of cell multiplication was in the order C3 > C2 > C1. That is corresponding to 25.28%, 35.92%, and 41.26%, respectively, evaluated at 100µg/ml (Fig. 6b).

### Cytotoxicity evaluation of quercetin and orange oil formulated in nanoemulsions

Figure 6a, b illustrates the behavior of cell proliferation percentage of HCT116 and Caco-2 cells as affected by treatment with micellar dispersion of quercetin (F1, F2), nanoemulsions of orange oil (F3, F4), and their mixture (F5, F6). The reader can bear in mind that (F1, F3, F5) were fabricated using a single surfactant, while (F2, F4, F6) were made using a mixture of surfactants as previously shown in Table 1.

The main results that can be concluded from the cytotoxicity study, which is represented in Fig. 6a, b are: First, phytochemical formulations (F1-F6) inhibit cell proliferation more than their corresponding natural unformulated counterparts, which were delivered in DMSO (C1-C3). Second, formulations made using a mixture of surfactants (F2, F4, F6) were more effective in inhibiting cell proliferation than those made using a single surfactant (F1, F2, F3). Third, Caco-2 cells were more sensitive toward the nanoemulsions compared to HCT116 cells. That is clearly manifested in Fig. 5b, where treatment of the cells with nanoemulsion (F6) led to almost zero percent proliferation.

#### Cytotoxicity evaluation in terms of IC_50_

Table 3 represents the IC_50_ values, which are a numerical expression of the cytotoxicity evaluation that was previously expressed graphically in details in the previous passages as cell proliferation percentage (Fig. 6a, b). Table 3 confirmed the results that we already discussed in the figures. For instance, the lowest IC_50_ value, (the highest anticancer activity) was 13.272 µg/mL, which corresponds to (F6), that is a nanoemulsion of a mixture of quercetin and orange oil formulated using a surfactant mixture against Caco-2 cell line.

**Table 3:** IC_50_ and fold change values of the different groups of micellar dispersion, nanoemulsions and controls against HCT116 and Caco-2 cells

Test groups	HCT 116 cells		Caco-2 cells	
	IC_50_ (µg/mL)	Fold change	IC_50_ (µg/mL)	Fold change
Group (1)				
C1	68.792	–-	62.888	–-
F1	50.144	1.372	43.583	1.443
F2	43.825	1.570	35.327	1.780
Group (2)				
C2	67.327	–-	61.391	–-
F3	55.469	1.214	47.314	1.298
F4	39.449	1.707	33.388	1.839
Group (3)				
C3	56.752	–-	50.534	–-
F5	26.788	2.119	18.227	2.772
F6	22.864	2.482	13.272	3.807

Fold change = IC_50 control_/IC_50 nanoemulsion_

C1-C3: controls (bioactives were dissolved in DMSO)

F1-F6: refer to footnote of Table 1

### Apoptotic evaluation using flow cytometric analysis

#### HCT116 cancer cell apoptosis

**Fig. 7:**
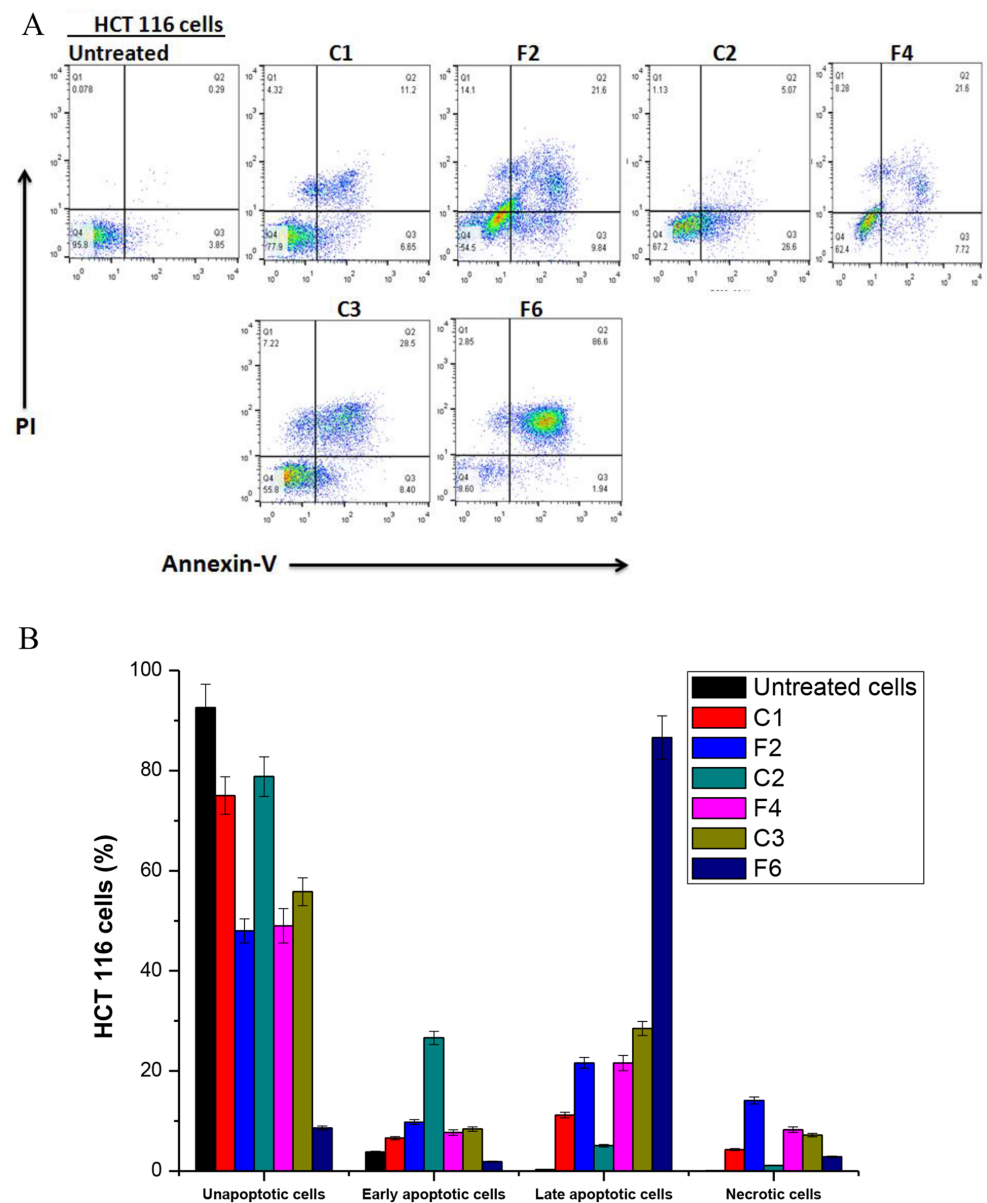
Apoptotic screening using flow cytometric analysis of the nanoemulsions (F2, F4, F6) against HCT116 cells. **a** Apoptotic diagrams; **b** Apoptotic graph (*n =* 3)

From Fig. 7a, it is evident that the count percent of colorectal cancer HCT116 cells in the quadrant of negative annexin V/negative PI was decreased gradually after treatment with F2, F4, and F6 compared to controls C1, C2, and C3, respectively. That indicates cancer cell viability decreased upon treatment with the three nanoemulsions, with the highest inhibition for F6 (quercetin-orange oil nanoemulsion).

Figure 7b shows 4 stages of cell death, namely, the un-apoptotic, early apoptotic, late apoptotic, and necrotic HCT116 cells as affected by the different treatments. In the first (un-apoptotic stage), the survival percentage of the HCT116 untreated cells that were left to grow freely without any treatment was 95.8%. The percentage decreased significantly to be 77.9%, 54.5%, 67.2%, and 62.4% after incubation with C1, F2, C2, and F4, respectively. Interestingly, F6 recorded the highest significant decrease in the unapoptotic HCT116 cells (8.60%) compared to its control C3 (55.8%) and the other formulations.

In the case of the second stage (early apoptosis), in which the programmed cell death starts, the percentage of the untreated HCT116 cells was 3.8% and began to increase significantly to be 6.6%, 9.8%, 26.6%, and 7.7%, after treatment with C1, F2, C2, and F4, respectively. On the other hand, F6 recorded the significant decrease in the early apoptotic HCT116 cells (1.9%) compared to its control sample C3 (8.4%).

Regarding the third late apoptotic stage, the untreated HCT116 cells was 0.29%, and the late apoptotic cells increased significantly to be 11.2%, 21.6%, 5.07%, and 21.6% after treatment with C1, F2, C2, and F4, respectively, while F6 recorded the highest significant increase in the late apoptotic HCT116 cells (86.6%) compared to C3 (28.5%).

Finally, in the necrotic stage, HCT116 untreated cells were 0.078% and the necrotic cells increased significantly to be 4.3%, 14.1%, 1.13%, and 8.28% after treatment with C1, F2, C2, and F4, respectively. On the other hand, F6 recorded the highest significant decrease in the necrotic HCT116 cells (2.85%) compared to C3 (28.5%).

#### Caco-2 cancer cell apoptosis

**Fig. 8:**
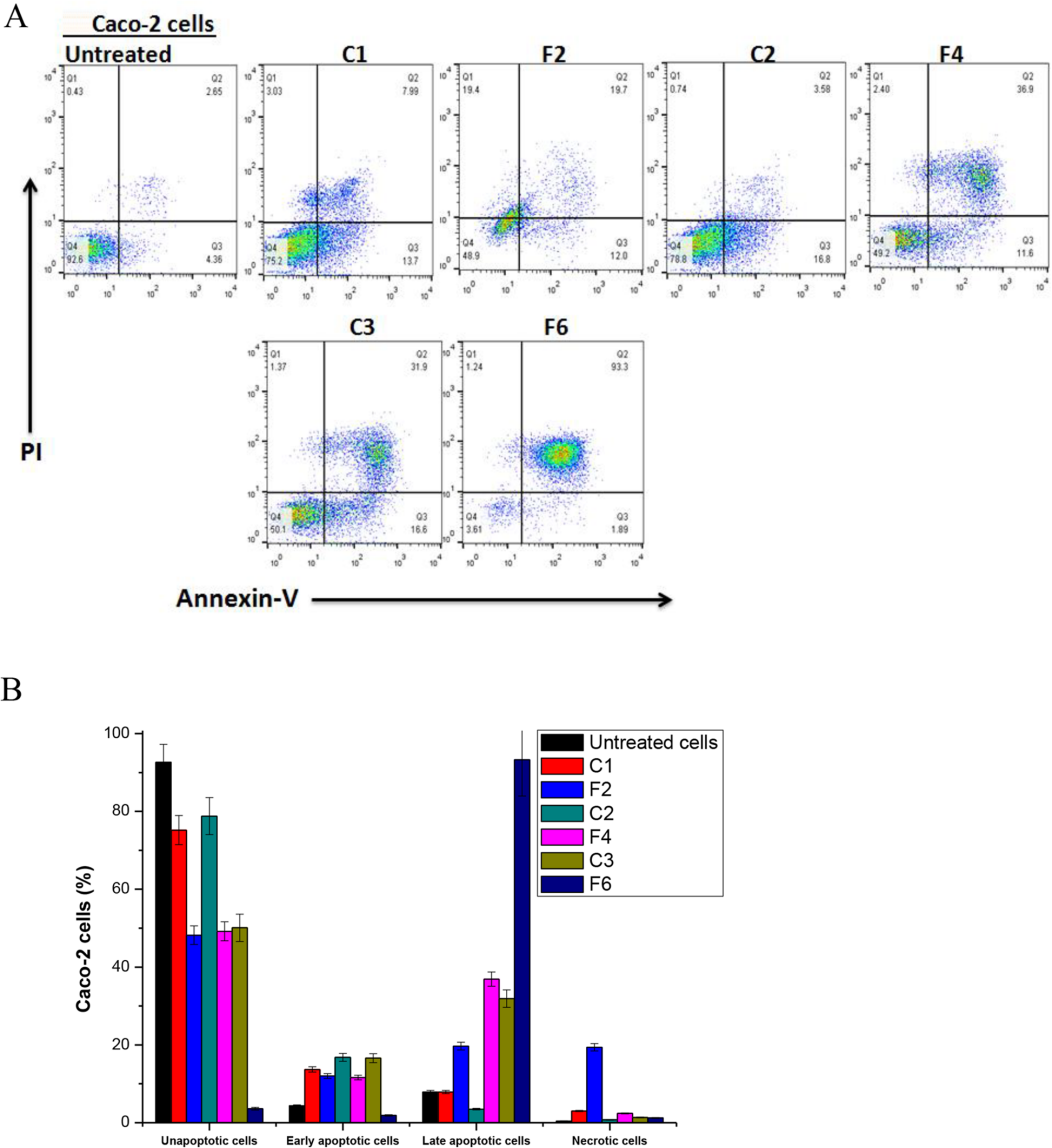
Apoptotic screening using flow cytometric analysis of the different nanoemulsions (F2, F4, F6) against Caco-2 cells. **a** Apoptotic diagrams; **b** Apoptotic graph (*n =* 3)

Similar to HCT116 cancer cell apoptosis, it was found that the count percent of Caco-2 cells in the quadrant of negative annexin V/negative PI were decreased gradually after treatment with formulations F2, F4, and F6 compared to controls C1, C2, and C3, respectively (Fig. 8a, b). Interestingly, F6 recorded the highest significant increase in the late apoptotic Caco-2 cells (93.3%) compared to its control C3 (31.9%). On the other hand, F6 recorded the lowest significant decrease in the necrotic Caco-2 cells (1.24%). This confirmed the potentials of nanoemulsions to enhance the apoptotic effect of the bioactive compound(s) compared to their natural unformulated states.

#### Evaluation of reactive oxygen species

**Fig. 9:**
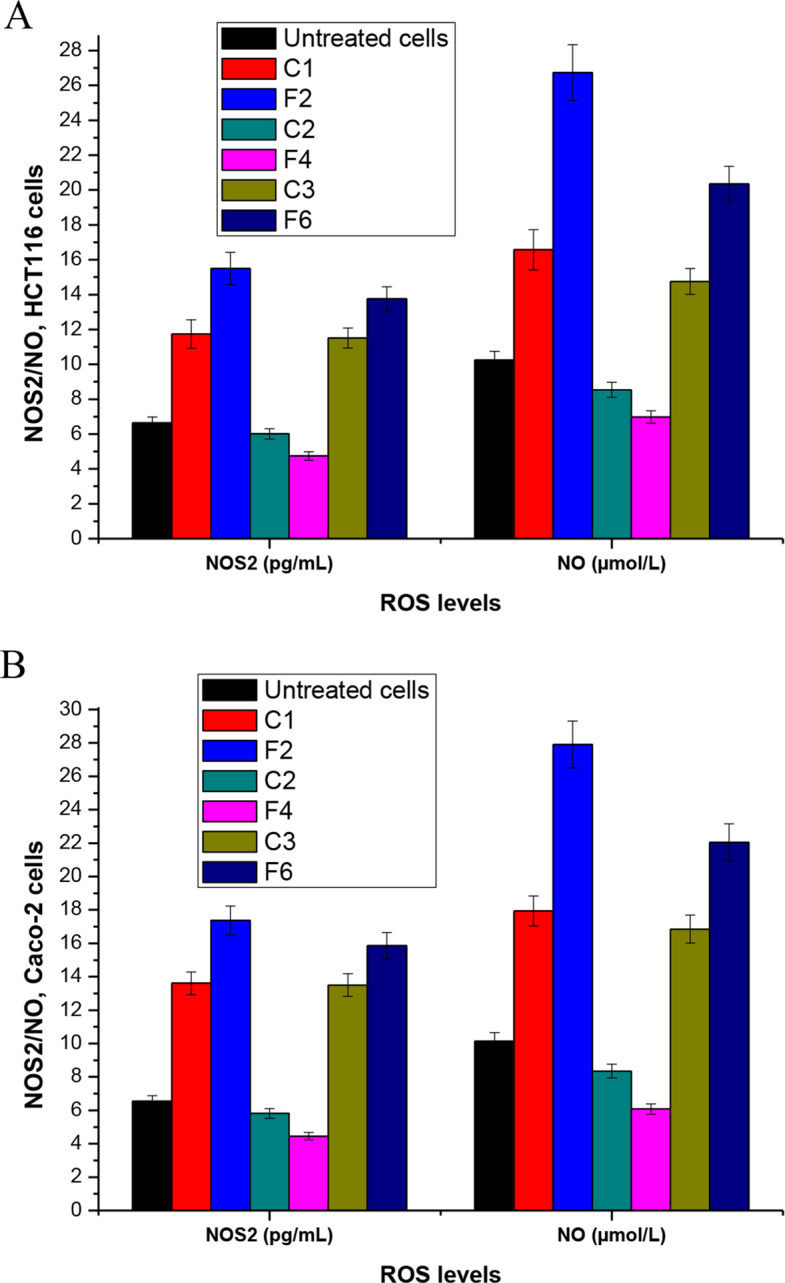
ROS measurements; NOS2 enzyme activity and NO level in **a**) colorectal HCT116 cells, and **b** colorectal Caco-2 cells (*n =* 3)

Figure 9a, b showed ROS measurements, including NO levels and NOS2 activity in colorectal HCT116 and Caco-2 cells. It was noted that quercetin in the unformulated form (C1) caused significant elevation (*P <* 0.05) of NO levels and NOS2 activity in both cancer cell lines. This significant elevation was augmented (*P <* 0.01) in the aqueous micellar form of quercetin (F2). Intriguingly, Caco-2 cells were more sensitive to NO and NOS2 elevation than HCT116 cells. NO and NOS2 levels reached the maximum upon treatment of Caco-2 cells with F2 at 27.91 µmol/L and 17.37 pg/mL compared to the untreated Caco-2 cells (10.15 µmol/L and 6.55 pg/mL), respectively (Fig. 9a, b).

On the contrary, unformulated orange oil (C2) insignificantly decreased the NO and NOS2 levels in both colorectal cancer cells; however, its nanoemulsion (F4) caused a decrease in the NO and NOS2 levels in both colorectal cancerous cells (Fig. 8a, b). Regarding the unformulated quercetin-orange oil mixture (C3), they can cause significant elevation (*P <* 0.05) of NO and NOS2 levels compared to the untreated HCT116 and Caco-2 cells. Formulation of this mixture in nanoemulsion (F6) can even enhance the values of the NO and NOS2 compared to the untreated HCT116 and Caco-2 cells (*P <* 0.01) (Fig. 9a, b).

#### Protein expression of PI3k, CDC25A, Bax and Bcl-2

**Fig. 10:**
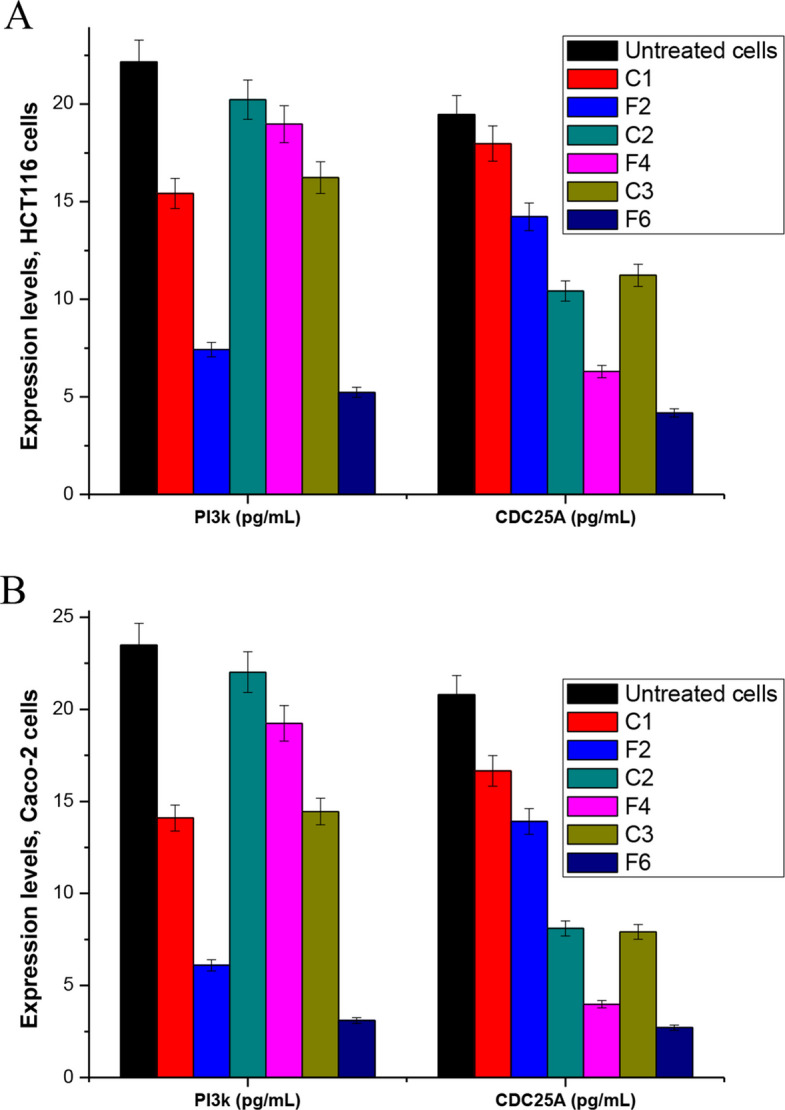
PI3k and CDC25A expression levels; PI3k and CDC25A expression levels in **a**) colorectal HCT116 cells, and **b** Caco-2 cells (*n =* 3)

Figure 10a, b indicated that PI3k and CDC25A were down-regulated in colorectal cancer HCT116 and Caco-2 cells upon treatment with all formulations. The highest inhibition of Caco-2 cells treated with quercetin-orange oil nanoemulsion (F6) was found.

**Fig. 11:**
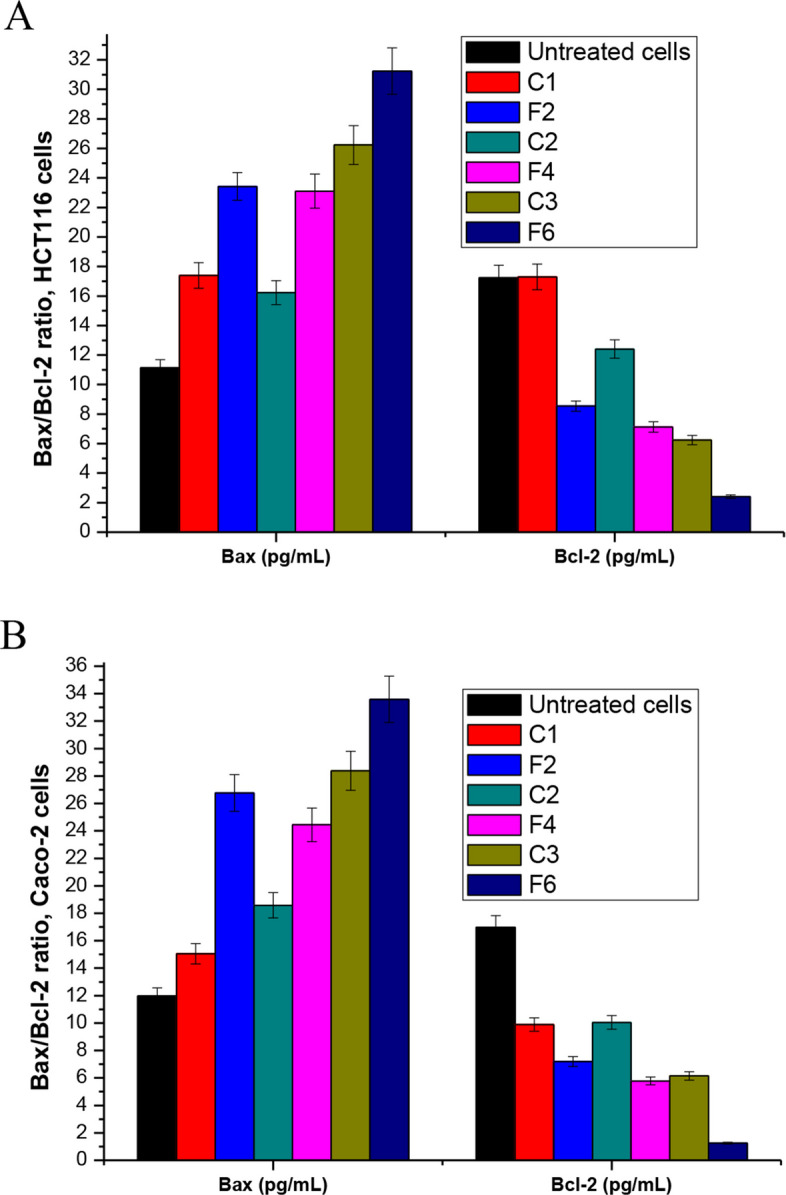
Bax and Bcl-2 expression levels. Bax and Bcl-2 expression levels in **a**) colorectal HCT116 cells, and **b** Caco-2 cells (*n =* 3)

In terms of the intrinsic mechanism of apoptosis, the activation of pro-apoptotic (Bax) and inhibition of anti-apoptotic (Bcl-2) are essential in the apoptotic response. Figure 11a, b demonstrate the expression levels of Bax and Bcl-2 proteins in both colorectal cancer cells. Caco-2 cells treated with nanoemulsion (F6) showed the highest Bax expression (33.59 pg/mL) and the lowest Bcl-2 level (1.25 pg/mL) compared to untreated cells (11.97 pg/mL and 16.98 pg/mL), respectively (Fig. 11b).

Table 4 indicates the Bax/Bcl-2 ratio, in which a ratio above 1.0 is an indication of apoptotic induction, while a ratio below 1.0 is an indication of apoptotic suppression. From the table, it is clear that all formulations (F2, F4, F6) and their controls (C1, C2, C3) recorded a Bax/Bcl-2 ratio higher than 1.0. This ratio was higher in the case of nanoemulsions F2, F4, and F6 compared to C1, C2, and C3. As usual, F6 recorded the highest Bax/Bcl-2 ratio (12.96 and 26.87) for HCT116 and Caco-2 cells, respectively.

**Table 4:** Bax/Bcl-2 ratio of micellar dispersion and nanoemulsions fabricated using surfactant mixture against HCT116 and Caco-2 cells

Bax/Bcl-2 ratio	Group (1)		Group (2)		Group (3)	
	Control (1)	F2	Control (2)	F4	Control (3)	F6
HCT116 cells	1.01	2.74	1.31	3.24	4.20	12.96
Caco-2 cells	1.52	3.72	1.85	4.23	4.61	26.87

F2, F4 and F6 and refer to footnote of Table 1

## Discussion

The two aqueous quercetin micellar dispersions subgroups F1(test) and F2(test), containing 500 mg quercetin, were fabricated using 5% surfactant(s) and 1% cosurfactant (PG) and water to 100 ml (Table 1). These percentages were chosen based on our previous investigations on the spontaneous fabrication of phytochemical nanoemulsions using the low-energy approach [15, 17, 18]. The chosen amount of surfactant(s) and cosurfactant of F1(test) and F2(test), in Table 1 were found to be appropriate for dissolving the amount of quercetin powder in a homogeneous concentrate. However, upon titration of that concentrate into water to form aqueous micellar dispersion, quercetin immediately precipitated (as shown in Fig. 2a. This indicates that the chosen amount of surfactant(s) and PG in subgroups F1(test) and F2(test) concentrates, is not sufficient for holding this compound stable as nanoparticles after titrating their concentrate into water to form the aqueous micellar dispersions. Therefore, the percentages of surfactants and cosurfactants were increased gradually till optimized at 10% and 2%, respectively. These percentages were found to be optimum for complete incorporation of quercetin in the both aqueous micellar dispersions (F1) and (F2) (Fig. 2b). The physical stability of (F1 and F2) was examined for 30 days; however, after only 1 week, formula (F2), which was made with a mixture of surfactants, was found to be unstable, and some quercetin precipitations were detected (Fig. 2c). On the other hand, formula (F1), which was made with a single surfactant, was stable during the week, indicating that only a single surfactant like Cremophor at 10% of the aqueous micellar dispersion has the necessary hydrophilic-lipophilic balance and affinity to quercetin to keep it totally solubilized in the system. The match of HLB of the surfactant with that of the solubilizate is an important factor for micellar solubilization and also for the formation of stable nanoemulsions. However, at the end of the 30-day storage period, few quercetin precipitations were detected in F1, as seen in Fig. 1a, bottom. It is worth indicating that the application of quercetin-containing formulas (F1, F2, F5 and F6), on the tested cancer cell lines took place within 24 h after the fabrication, where quercetin was completely incorporated within each formula, without any precipitations.

Before the fabrication of group (2), orange oil nanoemulsions (F3, F4), the oil was subjected to GC analysis, which revealed that d-limonene represents the major constituent at 89% of the oil composition. This finding came close to another study [19], which reported 88% d-limonene, while others [20] found that d-limonene comprises 95.5% of the oil composition. Although orange peel oil contained more terpene constituents beside limonene, we focused only on this compound in the discussion section. That is because d-limonene constitutes most of the oil composition, beside its inhibitory activity against colon cancer [20] and other types of cancer cells [19, 21, 22].

d-Limonene in the orange oil represent a challenge to the physical stability of its nanoemulsion due to the slight water solubility of this compound. This character induces Ostwald ripening destabilization mechanism [23], which leads to emulsion separation right after formulation. Nevertheless, orange oil nanoemulsions (F3 and F4), were physically stable for a 30-day storage period at 25℃ without detection of any oil separation (Fig. 1, bottom photo). That is due to the incorporation of a triglyceride, (sunflower oil) at 1% into orange oil (5%), as an Ostwald ripening inhibitor (Table 1), [15]. That reduces the chemical potential gradient and decreasing d-limonene migration from tiny particles, through water, to larger particles under the effect of Laplace pressure [23]. Additionally, nonionic surfactants (Cremophor RH40 and Tween 80) provided steric stabilization, preventing droplet coalescence even at low zeta potential (~ −1 mV). No detection of phase separation or oil creaming after 24 h even at 37 °C, and the nanoemulsions maintaining optical transparency. These findings confirm that the combination of the triglyceride and appropriate proportions of sterically stabilizing nonionic surfactants effectively prevent Ostwald ripening, and gravitational separation, leading to a stable nanemulsion.

It was interesting to note that at the end of the 30-days storage period, the yellow color of orange oil nanoemulsions (F3, F4) had become colorless, especially F4 (Fig. 1, bottom), with full retention of orange odor. The exact reason is yet to be revealed to us since this is the first time to report this phenomenon with the spontaneous fabrication of orange oil nanoemulsion. However, we can preliminarily postulate that the nanoemulsification is a process of continuous equilibration of ingredients over the storage period. That leads to more solubilization of orange oil nanoparticles that carry β-carotene and the other yellow coloring pigments. This dynamic process leads to much smaller nanoparticles that do not allow visible light to scatter from the nanoparticles.

Regarding group (3) nanoemulsions, quercetin was incorporated together with cold-pressed orange oil in the same formula (subgroups F5 and F6, Table 1). Unlike orange oil nanoemulsions (F3, F4), the yellow color of F5 and F6 was retained after storage, which is due to the intensive yellow color of the incorporated quercetin powder.

The particle size and zeta-potential of all formulation had a size in the nano-range (< 100 nm), with narrow size distribution (PDI 0.1), Fig. 3a and Table 2. The zeta-potential values were very small, approaching about −1.0 mV, which is expected due to the application of non-ionic surfactants in the current study. Therefore, the stability of nanoemulsion originate from the stearic hindrance mechanism of the bulky surfactants, rather than the charge repellence mechanism which required not less than ± 30 mV in order to be applied. TEM images (Fig. 3b) indicates that particles have spherical morphology with homogenous distribution. It is worth noting that the choice of F4 (quercetin-free orange oil nanoemulsion) and F6 (quercetin-loaded orange oil nanoemulsion) for TEM investigation is due to their high cytotoxic and apoptotic activity against the different cancer cell lines compared to the other formulations, as shown in the results section.

The high EE% observed across all formulations (> 93%, Table 2) indicates the strong potentials of the nanoemulsion systems to efficiently encapsulate both quercetin and orange oil. This can be attributed to the small droplet size (12.3nm–26.8 nm) and low PDI values (< 0.1), which reflect a uniform and stable system with a large interfacial area that enhances component’s incorporation. Generally using a mixture of two surfactant enhances the encapsulation efficiency due to the formation of mixed film surfactant around the encapsulated droplets.

Regarding the release of quercetin and orange oil from nanoemulsions, Fig. 5a, b showed that all nanoemulsion formulations exhibited a controlled, time-dependent release profile for both quercetin and orange oil. That is characterized by an initial moderate release within the first few hours followed by a sustained release up to 24 h. It is well known that in most liquid nanoemulsion systems, release follows zero-order kinetics when the oil must diffuse out of the droplet through the surfactant layer.

The activity of quercetin for inhibiting colorectal cancer cell proliferation takes place through regulating the expression of anti-aging gene SIRT-6 [1]. In a rat model of colorectal cancer, quercetin supplementation leads to an increase in apoptotic gene expression, including caspase 3, and a decrease in anti-apoptotic gene expression, including Bcl-2 [3]. On the other hand, other investigators [24] indicated that quercetin has the ability to inhibit the nuclear factor-Kappa B pathway (NF-κB), as well as down-regulate B-cell lymphoma 2 (Bcl-2) and up-regulate Bax. A detailed mechanism that rationalizes the anticancer activity of quercetin on colorectal and other organs was also discussed [25]. That includes regulation of signaling pathways like apoptotic, p53, p38 NF-κB, MAPK/Erk, JAK/STAT, PI3K/AKT, and Wnt/β-catenin. The same mechanisms of action of quercetin against colorectal cancer cells were also proposed [2].

The antiproliferative activity of natural unformulated orange peel oil in DMSO (C2) on both colon cancer cells (Fig. 6a, b) originates mainly from d-limonene, which is the main volatile constituent of orange peel oil (89%) as we found in the current investigation. An early investigation [20] concluded that d-limonene is responsible for the pro-apoptotic and anti-angiogenesis potential of orange oil on colorectal cancer cells. Later on, the antiproliferative activity of orange oil (88% d-limonene) against HCT116 colorectal cancer cells and its migration was also reported [19].

The mechanism of action was revealed using LS174T cells [26], where d-limonene induced apoptosis via the mitochondrial death pathway and the suppression of the PI3K/Akt signaling pathway. More mechanisms of action of d-limonene were recently illustrated in details elsewhere [21].

Combining quercetin with orange oil in DMSO (control C3, Table 1) led to an enhanced inhibitory activity against the proliferation of HCT116 colorectal cancer cells (Fig. 6a). That clearly demonstrates the synergistic inhibitory effect of a mixture of quercetin and orange oil on the proliferation of cancer cells.

Regarding Caco-2 colorectal cell line, the cytotoxicity of unformulated quercetin, orange oil, and their mixture (C1-C3) against these cells was illustrated in (Fig. 6b). The results showed the same trend that we described before for HCT116 cells. The noticeable difference between HCT116 cells (Fig. 6a) and Caco-2 cells (Fig. 6b) is that Caco-2 cells are more sensitive to the treatment with (C2) than HCT116 cells. That is clear from the lower cell proliferation percentage of Caco-2 cells compared to that of HCT116 cells.

In the current investigation, quercetin, orange oil, and their mixture were formulated in a water-based micellar dispersion and nanoemulsion delivery system for practical application in an aqueous environment or relevant administration routes. The previously mentioned results of Fig. 6a, b indicate that nanoemulsion can enhance the anticancer activity of the bioactive phytochemical, especially when formulated using a mixture of surfactants. That is due to better diffusion of nanoparticles into the cancer cells, as was proved in our previous work [18], after labeling nanoemulsions with Alexa Flour-488 dye followed by tracking the fluorescence in the cancer cells.

After studying the cytotoxic effect of the phytochemicals and their nanoemulsions against colorectal cancer cells, this section investigates the mechanistic approach for inducing these cells toward apoptosis. That approach was fulfilled through flow cytometric analysis, which is one of the common techniques used to study apoptosis. The cytotoxicity evaluation in the previous section of the current work included all sub-groups of formulations (F1-F6). However, in this section the investigation will focus only on the effect of three formulation sub-groups (F2, F4, and F6) on the apoptosis of the tested colorectal cancer cells. That is based on the previously shown enhanced activity of these sub-groups (Fig. 6a, b, Table 3), which were fabricated using a mixture of surfactants.

Figures 7, 8 reported the results of applying formulations F2, F4, and F6 for driving HCT116 and Caco-2 cancer cells toward apoptosis. The results obtained were in accordance with our former published work on *Nigella sativa* volatile oil nanoemulsion [18]. Some volatile oils have the ability to cause the mitochondrial membranes to depolarize, which can cause aberrant membrane permeability and the release of pro-apoptotic substances such as cytochrome c, radicals, and calcium ions, which in turn induce apoptosis [27]. Data obtained confirmed the potentials of nanoemulsions to enhance the apoptotic effect of the bioactive compound(s) compared to their natural, unformulated states.

Reactive oxygen species (ROS), which include nitric oxide (NO) and its enzyme nitric oxide synthase-2 (NOS2), can cause DNA damage and p53 activation of cancer cells, leading to Bax upregulation (i.e., intrinsic apoptotic pathway) [28]. Measurements of ROS markers were performed to investigate the role of formulations (F2, F4, and F6) on the induction of apoptosis.

The present results are in agreement with our previous work [29] where viramidine nanoemulsion could significantly raise NO through inducible nitric oxide synthase (iNOS) activation in hepatic cancer cells. This may be mediated by extrinsic and intrinsic apoptotic pathways. This strategy confirmed that the formulated nano-delivery system can down-regulate the c-FLIP, Bcl-2, and TNF-α expression levels and up-regulate FADD, caspase 8, caspase 3, caspase 9, and Bax expression levels.

In some cases of cancer treatment, chemo-resistance develops, leading to the reoccurrence of cancer due to the induction of some survival (anti-apoptotic) proteins such as PI3k and CDC25A. Thus, inhibition of these proteins is an attractive therapeutic strategy, which is targeted in the current study. Figure 10a, b indicated that PI3k and CDC25A were down-regulated in colorectal cancer HCT116 and Caco-2 cells upon treatment with all formulations.

It was suggested that cancer is associated with elevated levels of CDC25 isoforms A, B, and C. Remarkably, inhibiting CDC25C promoted programmed cell death. The proapoptotic Bcl-2-associated death promoter's inhibitory phosphorylation on amino acid Ser136 was raised by CDC25C. On the other hand, Akt's activating phosphorylation on amino acid Ser473 was elevated [30]. Previously, anticancer effect of some nanoparticles significantly enhanced cytotoxicity through the genetic down-regulation of STAT-1, NF-kB, and PI3k expression levels [30]. Inhibition of CDC25A and PI3k is critical for providing new strategic treatments against cancer. Parallel to this, it was found that nanoparticles inhibited the growth of cancerous cells by arresting the cell cycle and affecting the p53, PI3k and CDC25A expression levels [30]. Some nanoemulsions may possibly be cytotoxic to cancer cells in-vitro by modulating various genetic markers, including microRNA and long noncoding RNA. This strategy may facilitate cellular uptake of the nanoemulsions through CD44-expressed cancerous cells [30, 31].

Generally, essential oils were also demonstrated to change expression levels of Bcl-2 and Bax genes, leading to the release of cytochrome C and the induction of apoptosis in cancerous cells. This happens via activation of caspases 9 and 3, which in turn causes apoptosis. Antiapoptotic Bcl-2 protein is downregulated by the action of essential oil on the cancerous cells [32]. Quercetin [33] and d-limonene [26] (which is the main component of orange oil) significantly reduce the expression of the antiapoptotic protein Bcl-2 and induce proapoptotic Bax. This suggests sensitization of the cancerous cells toward apoptosis.

## Conclusions

Colorectal cancer requires continuous investigations to develop medications or adjuvants for the classical ones for better driving of the malignant cells toward apoptosis. This trend was approached in the current investigation using a combination of natural phytochemicals, including, quercetin and an cold-pressed orange peel oil mixture fabricated in specially designed water-based nanoemulsions. Nanoemulsion fabricated with a mixture of two surfactants showed better cytotoxic and apoptotic activity through downregulation of CDC25A, PI3k, and Bcl-2 expression levels and upregulation of Bax expression compared to nanoemulsion made with a single surfactant. The fabrication technique described in this work is facile and can be practically scaled up to industrial levels with economic feasibility. More investigations will be conducted in the future by our groups on animal models using the preliminary results obtained from the current study.

## Data Availability

Data is available upon request from the corresponding authors.
